# Intraoperative Neurophysiological Monitoring During Continuous Radiofrequency Treatment of Morton’s Neuroma: Complete Ablation

**DOI:** 10.3390/medicina62102025

**Published:** 2026-10-19

**Authors:** Gabriel Camuñas-Nieves, Alejandro Fernández-Gibello, Francisco Martínez-Pérez, Rubén Montes-Salas, Felice Galluccio, Alfonso Martínez-Nova

**Affiliations:** 1 Clínica Vitruvio, C/ María de Guzmán 47, 28003 Madrid, Spain; gabrielcamunas@gmail.com (G.C.-N.); alejandrofernandezgibello@gmail.com (A.F.-G.); fran.martinezperez@gmail.com (F.M.-P.); rumonsa@gmail.com (R.M.-S.); 2 Fisiotech Lab Studio, Rheumatology and Pain Management, 50136 Firenze, Italy; felicegalluccio@gmail.com; 3 Center for Regional Anesthesia and Pain Medicine (CRAPM), Wan Fang Hospital, Taipei Medical University, Taipei 116, Taiwan; 4 Department of Nursing, University of Extremadura, 10600 Plasencia, Spain

**Keywords:** Morton neuroma, radiofrequency, neurophysiological monitoring, neural ablation

## Abstract

*Background and Objectives*: Morton’s neuroma is a compressive neuropathy of the common plantar digital nerve that most frequently affects the third intermetatarsal space. Continuous radiofrequency (RF) ablation has reported success rates of approximately 80–85%; however, recent histological evidence suggests that RF does not consistently achieve complete neural ablation. This study aimed to evaluate the effectiveness of intraoperative neurophysiological monitoring of sensory nerve action potentials (SNAPs) to optimize the outcomes of continuous RF ablation in the treatment of Morton’s neuroma. *Materials and Methods*: This prospective case series included 22 patients (30 neuromas) treated with ultrasound-guided continuous RF ablation combined with intraoperative neurophysiological monitoring. SNAPs were recorded before, during, and after each RF application until complete abolition of sensory nerve conduction was confirmed. Clinical outcomes were assessed using the Visual Analog Scale (VAS), the Foot Function Index (FFI), and the Giannini Interdigital Neuroma Clinical Evaluation Score at 3, 6, and 12 months postoperatively. *Results*: At the 12-month follow-up, satisfactory outcomes (Giannini score ≥60) were achieved in 86.7% of neuromas (26/30). The mean VAS pain score decreased from 8.67 preoperatively to 1.67 at 12 months. Among the seven neuromas with previous unsuccessful RF treatment, all achieved satisfactory outcomes following the monitored procedure. The four cases with poor outcomes were predominantly associated with a positive anesthetic suppression test, suggesting concomitant mechanical metatarsalgia rather than treatment failure. After excluding cases with likely diagnostic misclassification, the adjusted success rate increased to 96.3%. *Conclusions*: Intraoperative neurophysiological monitoring enables real-time confirmation of complete sensory nerve ablation during continuous RF treatment. Its main potential value appears to lie in patients with previous unsuccessful conventional RF, all of whom achieved satisfactory outcomes in this series. Persistent postoperative symptoms appeared to be more closely related to concomitant forefoot pathology than to technical limitations of the RF procedure.

## 1. Introduction

Morton’s neuroma is a chronic compressive and degenerative neuropathy of the common plantar digital nerve, characterized histopathologically by perineural and endoneural fibrosis rather than a true neuroma, and most commonly affects the third intermetatarsal space, followed by the second [1,2]. Diagnosis relies on the correlation between clinical findings and imaging, particularly ultrasonography and magnetic resonance imaging, although imaging abnormalities do not always correlate with symptoms and may coexist with other causes of forefoot pain, such as mechanical metatarsalgia, intermetatarsal bursitis, or plantar plate injury [3,4,5,6].

Surgical neurectomy through a dorsal approach remains the standard treatment for patients who fail conservative management [7], with good or excellent outcomes reported in 51–76.5% of cases. Nevertheless, persistent pain and stump neuroma formation remain important drawbacks, in addition to the expected permanent sensory loss in the interdigital territory, which may be bothersome for some patients [8,9]. Consequently, continuous radiofrequency (RF) ablation has emerged as a minimally invasive alternative [10,11]. By delivering high-frequency alternating current through an electrode, continuous RF produces a controlled thermal lesion that induces coagulative necrosis of neural tissue while avoiding open surgery [12]. Clinical studies have reported success rates ranging from 80% to 87%, based on broader clinical outcome criteria such as patient satisfaction or partial-to-complete symptom improvement, whereas a recent systematic review and meta-analysis demonstrated complete pain relief, a more stringent outcome, in approximately 47.6% of patients with superior outcomes achieved using temperatures of at least 85 °C [11,13,14,15].

Despite these encouraging results, continuous RF has important technical limitations. Experimental models by Cosman et al. have shown that the thermal lesion generated by a conventional RF electrode (SMK-C10, 5-mm active tip) reaches approximately 4 mm in transverse width within 60–120 s, with minimal further growth thereafter [12]. Previous ultrasonographic studies have reported mean Morton’s neuroma diameters of approximately 5–6 mm, with reported ranges extending from 3 to 10 mm [16,17]. Consequently, larger neuromas may not be completely ablated by a single application, which may explain why multiple applications often provide superior clinical outcomes and why neuroma size has been identified as a potential predictor of treatment failure [15,18].

Independently, our previous histological study in Morton’s neuromas, performed using continuous RF at 90 °C for 90 s with a 5-mm monopolar active tip, showed a homogeneous central area of coagulative necrosis with a maximum necrosis depth of approximately 2.4–3.2 mm, while viable neural tissue persisted beyond the necrotic area [19]. Although a 10-mm monopolar active tip was used in the present study, following the clinical protocol described by Masala et al. [15], the experimental models of Cosman et al. show that increasing active-tip length mainly increases the longitudinal extent of the lesion, with a comparatively smaller increase in transverse width [20]. Moreover, considerable variability in lesion size has been observed despite identical treatment parameters, suggesting that differences in neuroma size and fibrotic composition may influence thermal energy distribution and limit complete neural destruction. These findings indicate that incomplete nerve ablation may account for a substantial proportion of the reported treatment failures [19,20,21].

Current RF protocols are therefore based on a predefined number of applications without any objective confirmation that neural conduction has been abolished. Intraoperative neurophysiological monitoring may overcome this limitation by providing real-time functional assessment of the treated nerve. Recording nerve action potentials (NAPs) is considered the reference standard for evaluating nerve continuity during peripheral nerve surgery and guiding intraoperative decision-making [22]. Similarly, sensory nerve action potentials (SNAPs) have been extensively used for the diagnosis of interdigital neuropathy. Axonal degeneration and intraneural fibrosis produce a marked reduction in SNAP amplitude and conduction velocity, yielding reported diagnostic sensitivities of 77–93% [23,24,25]. In the foot, SNAPs can be obtained by stimulation of the tibial nerve at the ankle and recording from the toes corresponding to the evaluated interdigital space. Because SNAPs directly reflect the functional integrity of sensory nerve fibers, their disappearance following RF treatment could provide objective evidence of complete neural ablation.

Applying neurophysiological monitoring in the setting of intentional nerve ablation represents a conceptual shift. Rather than preserving neural conduction, the objective is to confirm its complete interruption. Persistent SNAPs after RF application would indicate residual viable neural tissue and incomplete ablation, whereas the absence of SNAPs would confirm successful functional denervation. To our knowledge, no previous study has evaluated intraoperative SNAP monitoring as a real-time feedback tool during continuous RF ablation for Morton’s neuroma, despite growing evidence that incomplete ablation is a major contributor to treatment failure [18,19]. Our hypothesis is that real-time monitoring of sensory nerve action potentials (SNAPs) during continuous RF would allow objective confirmation of complete interruption of sensory nerve conduction, thereby improving the effectiveness of the procedure compared with conventional RF protocols, which rely on a predetermined number of ablation cycles without functional confirmation. Therefore, the aim of this study was to evaluate the efficacy and safety of intraoperative neurophysiological monitoring of SNAPs as an objective real-time guidance tool to optimize continuous radiofrequency ablation in the treatment of Morton’s neuroma.

## 2. Materials and Methods

### 2.1. Study Design and Ethical Approval

**Figure 1:**
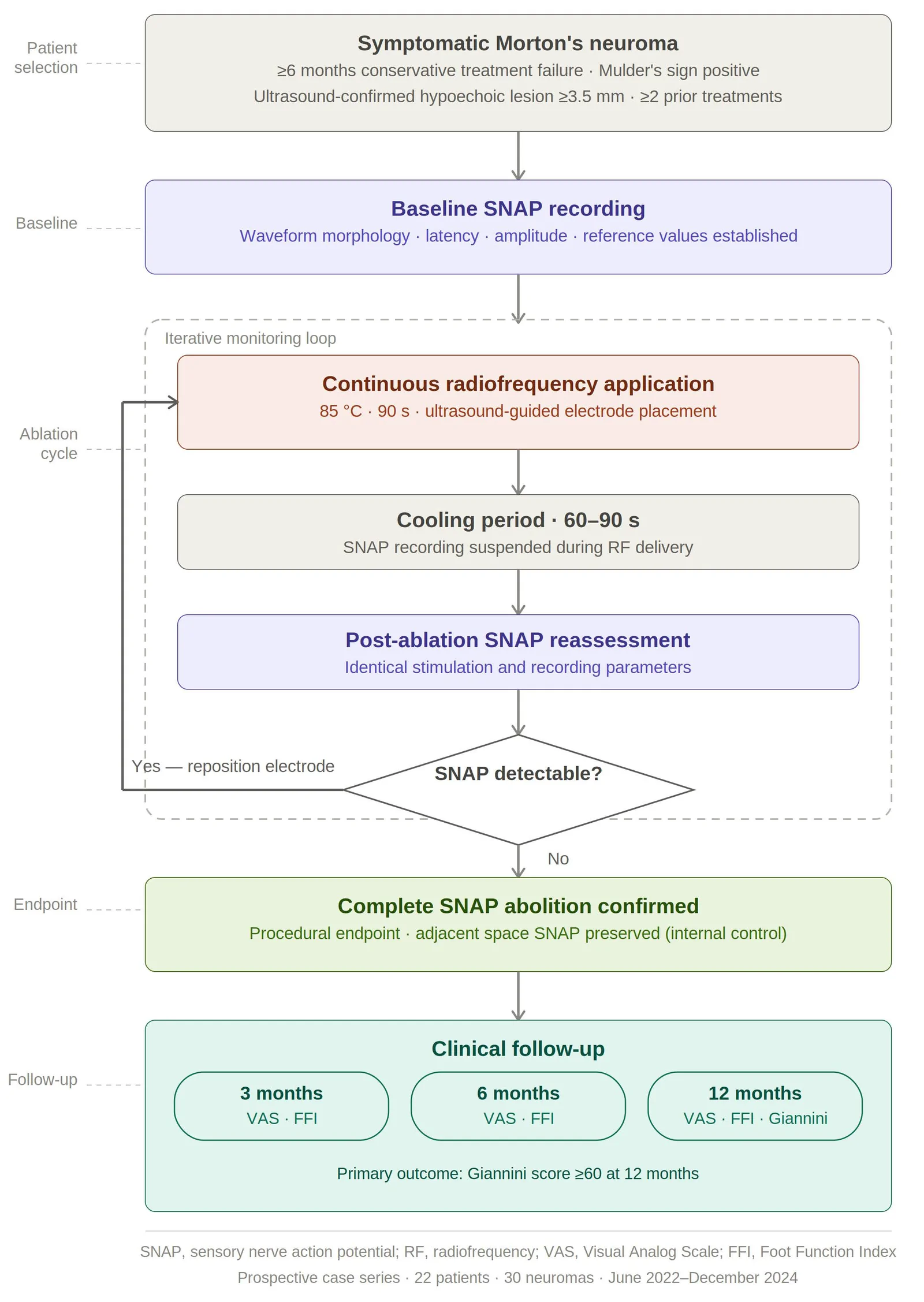
Overview of the study design and the SNAP-guided radiofrequency treatment protocol.

A prospective observational case series was conducted to evaluate the efficacy and safety of continuous RF ablation combined with intraoperative neurophysiological monitoring for the treatment of Morton’s neuroma. Consecutive patients were recruited between June 2022 and December 2024 and completed a minimum follow-up duration of 12 months. The study was conducted at Clínica Vitruvio (Madrid, Spain) in a minor surgery operating room. Radiofrequency procedures were performed by two experienced foot and ankle surgeons, while intraoperative neurophysiological monitoring was performed by a neurophysiology specialist. The study protocol was approved by the Research Ethics Committee of the University of Extremadura (Protocol No. 97/2022; approved on 16 April 2022). All participants provided written informed consent before enrollment, and the study was conducted in accordance with the principles of the Declaration of Helsinki. An overview of the study design and the SNAP-guided radiofrequency treatment protocol is shown in Figure 1.

### 2.2. Participants

22 (mean age 53.9 ± 15.5 years, range 31–76) adult patients (≥18 years) with symptomatic Morton’s neuroma, who had failed at least six months of conservative treatment were consecutively enrolled. The inclusion criteria were (a) clinical diagnosis was based on typical neuropathic forefoot pain, (b) localized tenderness within the affected intermetatarsal space, (c) positive Mulder’s sign, (d) ultrasonographic confirmation of an interdigital hypoechoic lesion measuring at least 3.5 mm in transverse diameter, (e) patients who had at least two previous Morton’s neuroma treatments, such as footwear modification, custom foot orthoses, ultrasound-guided corticosteroid injections, physiotherapy, previous continuous RF, or surgical decompression of the deep transverse intermetatarsal ligament. Patients were excluded if they presented contraindications to radiofrequency treatment, previous neurectomy in the same intermetatarsal space, peripheral neuropathies or other neurological disorders potentially affecting nerve conduction studies, active forefoot disorders that could interfere with clinical assessment, known allergy to amide local anesthetics, or inability to complete the follow-up protocol [3,5,26].

A total of 22 consecutive patients with 30 symptomatic neuromas were included. Each neuroma was considered the unit of analysis, consistent with the methodology adopted in previous studies of Morton’s neuroma. Although some patients contributed more than one neuroma, this analytical approach has been commonly used in previous case series evaluating treatment outcomes.

### 2.3. Continuous Radiofrequency Ablation Procedure

All procedures were performed on an outpatient basis under ultrasound guidance using a LOGIQ S7 ultrasound system (GE Healthcare, Wauwatosa, WI, USA) equipped with a high-resolution linear transducer (12–18 MHz). The neuroma was identified sonographically as a fusiform hypoechoic enlargement of the common plantar digital nerve located distal to the deep transverse intermetatarsal ligament. Its location and maximum transverse diameter were documented before treatment to plan electrode placement. Local anesthesia was achieved by an ultrasound-guided tibial nerve block using 1.5 mL of 1% lidocaine without epinephrine [27]. Subsequently, the radiofrequency electrode was introduced through the corresponding interdigital space under continuous ultrasound guidance and aligned longitudinally with the common plantar digital nerve. The entire 10-mm active tip was positioned over the healthy nerve segment immediately proximal to the neuroma, ensuring that the active tip remained completely outside the neuroma. Continuous radiofrequency was delivered using a dedicated radiofrequency generator (TLG10 RF Generator, TOP Medikey, Gouda, The Netherlands) equipped with a 10-mm active-tip radiofrequency electrode (TOP Medikey, Gouda, The Netherlands) at 85 °C for 90 s per application, according to previously published protocol [15]. A single monopolar electrode was used for each ablation cycle; no electrode array was used. The number of applications was not predefined but was individualized according to the intraoperative neurophysiological findings.

Following each ablation cycle, a cooling period of 60–90 s was allowed before reassessing sensory nerve conduction. Whenever SNAPs remained detectable, the electrode was repositioned under ultrasound guidance toward the region of persistent neural conduction, and an additional ablation cycle was performed. This iterative process continued until complete abolition of sensory nerve conduction was confirmed or until further treatment was considered unsafe because of the proximity of adjacent anatomical structures.

### 2.4. Intraoperative Neurophysiological Monitoring

Intraoperative neurophysiological monitoring was performed throughout the procedure using a dedicated electromyography system (Neuron-Spectrum-5S, Neurosoft Ltd., Ivanovo, Russia). Sensory nerve action potentials (SNAPs) were elicited by electrical stimulation of the tibial nerve at the ankle using self-adhesive surface electrodes and recorded using subdermal needle electrodes placed on the corresponding interdigital toes. Stimulation and recording parameters were adjusted to obtain reproducible supramaximal sensory responses according to established protocols for plantar digital nerve conduction studies [23,28]. The monitoring protocol consisted of three sequential stages. First, baseline SNAPs were recorded immediately before RF to confirm adequate signal quality and establish reference values for waveform morphology, latency, and amplitude. During each RF application, neurophysiological recording was temporarily suspended to avoid electrical interference generated by the radiofrequency current. Following completion of each ablation cycle and the corresponding cooling period, SNAPs were recorded again using the same stimulation and recording parameters. If SNAPs remained detectable, the electrode was repositioned under ultrasound guidance toward the area where residual sensory nerve conduction was considered most likely to persist, and an additional RF application was performed. This sequence was repeated until complete abolition of SNAPs was achieved, indicating complete interruption of sensory nerve conduction. Following confirmation of complete SNAP abolition in the treated interdigital space, SNAPs were recorded from the adjacent untreated interdigital space to confirm preserved sensory nerve conduction, which served as an internal control of the procedure.

### 2.5. Outcome Measures

The primary outcome was the clinical result at 12 months, assessed using the Giannini Interdigital Neuroma Clinical Evaluation Score (Giannini score) [29]. Treatment success was defined as a Giannini score of ≥60, corresponding to a good or excellent clinical outcome. Secondary outcomes included pain intensity, foot function, procedural variables, and treatment-related complications. Pain was assessed using a 10-point Visual Analog Scale (VAS), while functional status was evaluated with the validated Spanish version of the Foot Function Index (FFI) [30]. Both instruments were administered preoperatively and at 3, 6, and 12 months after treatment.

Procedural variables included the number of RF applications required to achieve complete SNAP abolition, neurophysiological monitoring findings, and intraoperative or postoperative complications. Patients with an unsatisfactory clinical outcome (Giannini score < 60) at the final follow-up underwent an ultrasound-guided diagnostic suppression test consisting of selective infiltration of the adjacent metatarsophalangeal joint with 2% mepivacaine. Pain relief greater than 50% was considered suggestive of an alternative mechanical source of pain rather than persistent neuropathic symptoms, thereby assisting in the interpretation of treatment failure and guiding subsequent management [31].

### 2.6. Follow-Up and Statistical Analysis

Patients were evaluated at 3, 6, and 12 months after the procedure. Continuous variables were expressed as the mean ± standard deviation (SD) or the median and interquartile range (IQR), depending on data distribution, whereas categorical variables were summarized as frequencies and percentages. Normality for changes in pain and functional outcomes (VAS and FFI scores) was assessed using the Shapiro–Wilk test and visual inspection of distribution plots. As the test indicated that the data were not normally distributed (*p* < 0.05), paired comparisons were performed using the Wilcoxon signed-rank test from baseline to 3, 6 and 12 months. An exploratory analysis was also performed to evaluate potential factors associated with treatment outcome, including patient demographics, neuroma characteristics, previous treatments, and the number of RF applications required to achieve complete SNAP abolition. Statistical significance was set at a two-sided *p* < 0.05. Statistical analyses were performed using IBM SPSS Statistics version 26.0 Campus License UEX (IBM Corp., Armonk, NY, USA).

## 3. Results

Twenty-two patients with a total of 30 Morton’s neuromas were consecutively included and treated with ultrasound-guided continuous radiofrequency and intraoperative neurophysiological monitoring. The baseline characteristics of the study population are summarized in Table 1. All patients had previously undergone conservative treatment for at least 6 months, including footwear modifications, foot orthoses, and physical therapy, without clinical improvement. In addition, the neuromas had previously been treated with one or more invasive procedures, including ultrasound-guided corticosteroid injections (*n* = 26), continuous radiofrequency (*n* = 7), and surgical release of the deep transverse intermetatarsal ligament (*n* = 2).

According to the study protocol, the number of radiofrequency applications was individualized according to the findings obtained through neurophysiological monitoring. The median number of applications required to achieve complete abolition of sensory nerve action potentials (SNAPs) was 3 (IQR: 2–4; range: 1–11), with complete abolition of SNAPs achieved in all 30 treated neuromas.

**Figure 2:**
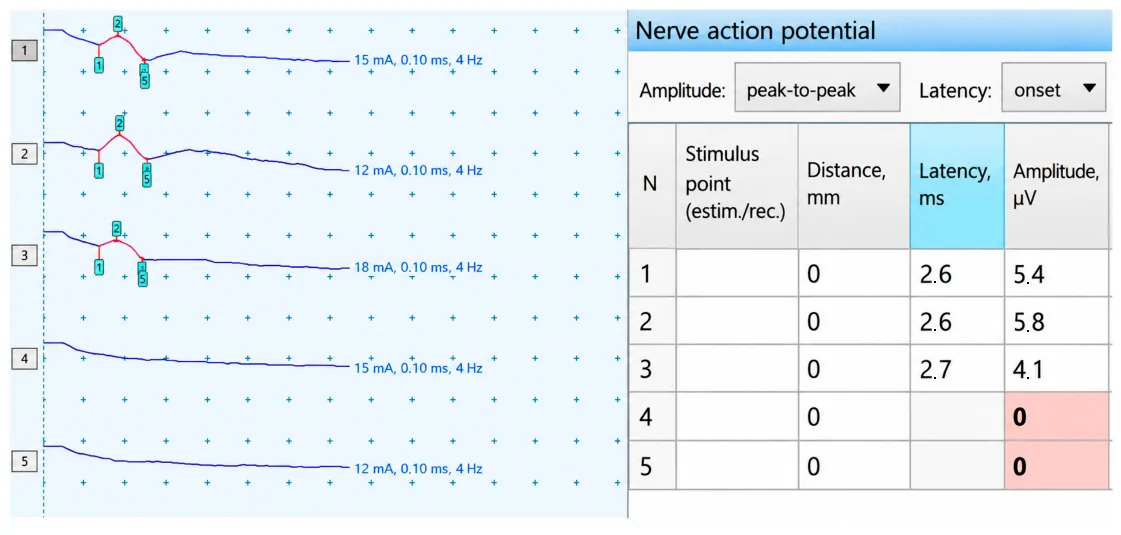
Representative intraoperative SNAP recordings in a patient with previous failed conventional RF. Trace 1: baseline SNAP; traces 2 and 3: after the first and second RF applications; trace 4: SNAP abolition after third RF; trace 5: confirmation of persistent SNAP absence.

A representative neurophysiological recording from a neuroma previously treated unsuccessfully with conventional RF is shown in Figure 2. Despite previous RF treatment, a recordable baseline SNAP was still present. During the monitored procedure, sensory conduction persisted after the first and second RF applications and was subsequently abolished, with absence of a recordable SNAP confirmed in two consecutive recordings.

Clinical outcomes during follow-up are shown in Table 2. The mean Giannini score at 12 months was 68.86 ± 18.53 points, with 26 of the 30 neuromas (86.7%) achieving a score ≥60. All seven neuromas previously treated unsuccessfully with conventional continuous RF achieved a satisfactory outcome (Giannini score ≥ 60) at 12 months following the neurophysiologically monitored procedure. Both VAS and FFI scores improved significantly compared with preoperative values. Mean VAS pain scores decreased significantly from 8.67 ± 1.35 preoperatively to 1.03 ± 1.43 at 3 months, to 0.93 ± 1.57 at 6 months, and to 1.67 ± 2.86 at 12 months (all *p* < 0.05 from the baseline, Table 2). Mean FFI scores decreased from 42.3 ± 13.4% preoperatively to 5.0 ± 7.4% at 6 months and to 8.0 ± 16.2% at 12 months (*p* < 0.001 and *p* < 0.05 from baseline, Table 2).

Four neuromas (13.3%) achieved a Giannini score below 60. Three of these underwent a diagnostic suppression test at the final follow-up, which was positive and enabled concomitant mechanical metatarsalgia to be identified as the source of residual pain. The fourth case developed persistent neuritis after the procedure. After excluding the three cases with residual pain attributable to concomitant mechanical metatarsalgia, 26 of the remaining 27 neuromas achieved a Giannini score ≥60, corresponding to an adjusted rate of favorable outcomes of 96.3%. No other major procedure-related complications were recorded during the follow-up period.

## 4. Discussion

This study evaluates the efficacy and safety of intraoperative SNAP monitoring during continuous radiofrequency treatment of Morton’s neuroma, with particular interest in patients with previous unsuccessful conventional RF treatment.

The main finding of the present study was that ultrasound-guided continuous radiofrequency with intraoperative neurophysiological monitoring achieved a clinical success rate of 86.7% at 12 months, with complete abolition of sensory nerve action potentials (SNAPs) achieved in all treated neuromas. In addition, neurophysiological monitoring enabled the number of applications required for each neuroma to be individualized and, after excluding patients with residual pain attributable to concomitant mechanical metatarsalgia, the adjusted success rate increased to 96.3%. These findings suggest that the combination of neurophysiological monitoring and diagnostic reassessment using the suppression test allows the clinical outcomes of continuous radiofrequency to be interpreted more accurately.

Our results are comparable to those reported in the main series of continuous radiofrequency treatment, with published success rates ranging from 83% to 89%, as well as to the findings reported in the meta-analysis by Llombart-Blanco et al., although the latter demonstrated substantial heterogeneity in inclusion criteria, treatment protocols, and outcome measures [11]. A comparison of the present study with the main published radiofrequency series is presented in Table 3.

It is noteworthy that the highest success rates corresponded to studies with follow-up periods of approximately six months. Similarly, in the series by Masala et al., mean VAS scores reached their lowest point at 6 months (1.7 ± 0.8) before rising again by 12 months (2.0 ± 0.4) [15]. In our series, patients with concomitant mechanical metatarsalgia experienced marked clinical improvement during the first six months, followed by progressive deterioration between six and twelve months [13,34]. Although this observation should be interpreted with caution and does not demonstrate a causal relationship, it suggests that longer follow-up may provide a more accurate estimate of medium-term clinical outcomes. From a mechanistic perspective, late changes in clinical outcomes could theoretically be related to axonal regeneration from surviving nerve fibers or collateral sprouting from adjacent nerves. However, this hypothesis cannot be confirmed from the present data, as longitudinal neurophysiological recordings were not performed during follow-up. Moreover, three of the four neuromas with an unfavorable outcome at 12 months had a positive suppression test, suggesting a concomitant mechanical source of pain rather than necessarily representing recovery of sensory nerve conduction.

Although the success rate achieved was similar to that reported in previous series, the main contribution of this study lies in the use of intraoperative neurophysiological monitoring. Unlike conventional radiofrequency protocols, this approach enabled the objective confirmation of complete abolition of SNAPs and allowed the number of applications required for each neuroma to be individualized. Although the median number of applications was three, some cases required up to eleven applications to achieve complete abolition of nerve conduction. The high number of applications required in some cases should not necessarily be interpreted as a consequence of greater neuroma diameter or length, as the number of applications was determined by persistence of sensory conduction rather than ultrasonographic size. It may instead reflect the spatial relationship between the electrode, the nerve, and the effective ablation volume, as well as variations in electrode positioning. These findings support the observations of Brooks et al. [18], who suggested that incomplete ablation may constitute a cause of treatment failure, and are consistent with the biophysical models described by Cosman [20], which demonstrated that the size of the thermal lesion is limited [13,14,15,18,20,35]. Furthermore, our previous histological study demonstrated the persistence of viable neural fascicles after a single radiofrequency application, providing a biological rationale for the need to individualize treatment according to the neurophysiological response of each neuroma [19,21].

From a logistical perspective, intraoperative SNAP monitoring and neurophysiological reassessment between RF cycles may increase the overall procedure time compared with conventional protocols, particularly in cases requiring multiple applications. However, total procedure time was not prospectively recorded as a standardized variable in the present study, precluding a formal comparison with previously published protocols.

One of the most relevant aspects of the present study was the analysis of patients with an unfavorable clinical course. Three of the four neuromas with a Giannini score below 60 had a positive suppression test, enabling concomitant mechanical metatarsalgia to be identified as the source of residual pain. These patients showed substantial improvement in VAS and FFI scores during the first six months, followed by progressive deterioration at one year, whereas the only patient with a negative suppression test exhibited a completely different clinical course, consistent with persistent neuritis. These findings suggest that some cases initially considered treatment failures did not actually represent failure of neuroma ablation but rather the coexistence of another source of forefoot pain. This interpretation is further supported by our previous anatomical study, which demonstrated that infiltration of the intermetatarsal space may simultaneously block the interdigital nerve and the metatarsophalangeal joint, thereby complicating the differential diagnosis of residual pain [31].

Another finding of interest was the favorable clinical course observed in the seven neuromas previously treated with conventional continuous radiofrequency. All achieved satisfactory outcomes following monitored radiofrequency treatment. Although the small sample size precludes definitive conclusions, these findings suggest that neurophysiological monitoring may represent a useful strategy in patients with previous failure of conventional radiofrequency treatment and support the hypothesis that incomplete ablation may account for some of these treatment failures [19].

Although the present study does not establish that SNAP monitoring improves clinical success rates compared with conventional RF, it provides an objective functional endpoint to confirm abolition of sensory conduction and individualize the number of RF applications. SNAP monitoring may be particularly useful when conduction persists after initial applications or in patients with previous RF failure.

Neurectomy remains the reference treatment for Morton’s neuroma refractory to conservative management, with published success rates ranging from 51% to 76.5% [8,9]. Neurectomy results in an expected permanent sensory deficit in the corresponding interdigital nerve territory, which may be bothersome for some patients. Other potential complications include stump neuroma, infection, painful scarring, and persistent neuropathic symptoms [8,9]. Continuous RF, as a neuroablative technique, could also produce a permanent localized sensory deficit in the territory of the treated nerve. Indeed, as with neurectomy, the therapeutic goal of continuous RF is permanent interruption of sensory conduction, since incomplete ablation or subsequent axonal regeneration could allow symptoms to recur; this provides the rationale for confirming SNAP abolition intraoperatively. In our series, ultrasound-guided continuous radiofrequency with neurophysiological monitoring achieved satisfactory clinical outcomes. Its potential added value may lie in the objective confirmation of sensory conduction abolition, particularly in patients with previous unsuccessful conventional radiofrequency treatment. Furthermore, Mann and Reynolds [2] reported that the deep transverse intermetatarsal ligament may heal and reattach following transection, suggesting that techniques based exclusively on ligament release may provide only temporary relief in some patients [2,36,37]. Pulsed radiofrequency has also demonstrated favorable clinical outcomes in at least one published series; however, its mechanism of action is predominantly neuromodulatory rather than ablative and therefore represents a therapeutic strategy distinct from continuous radiofrequency [33]. In our experience, phenol infiltration may constitute a salvage treatment option in selected cases with persistent neuropathic pain [38].

From a clinical perspective, our findings support the use of neurophysiological monitoring as an intraoperative control tool that enables the number of radiofrequency applications to be individualized and provides objective confirmation of the abolition of nerve conduction, particularly in patients with previous unsuccessful conventional RF, in whom incomplete ablation is a plausible cause of failure. Furthermore, the systematic use of the suppression test in patients with persistent pain may facilitate the identification of concomitant mechanical pathology and prevent some cases from being incorrectly interpreted as treatment failures related to the neuroma.

This study has several limitations. It was a prospective single-center series with a limited sample size and no control group treated with conventional radiofrequency. A potential selection bias should also be acknowledged. Consequently, although our findings support the potential utility of neurophysiological monitoring, they do not demonstrate that objective confirmation of SNAP abolition alone translates into a higher clinical success rate compared with conventional protocols. Prospective comparative studies are required to confirm these findings and to define the role of neurophysiological monitoring in the treatment of Morton’s neuroma.

## 5. Conclusions

The incorporation of intraoperative neurophysiological monitoring into continuous radiofrequency treatment for Morton’s neuroma may help to overcome a limitation of conventional protocols based on a predetermined number of applications. This approach individualizes treatment according to the neurophysiological response obtained during the procedure and provides an objective functional endpoint based on SNAP abolition. The clinical outcomes observed were within the range reported in previous series, although the absence of a control group does not allow superiority over conventional protocols to be established. Its main potential value may lie in patients with previous RF failure, as suggested by the satisfactory outcomes observed in all seven previously treated neuromas. Finally, appropriate patient selection, based on a rigorous diagnostic assessment that includes the suppression test, is important to maximize clinical outcomes and establish realistic expectations, particularly in patients with concomitant forefoot pathology.

## Figures and Tables

**Table 1:** Baseline demographic and morphological variables.

Variable	Value
Patients, *n*	22
Neuromas, *n*	30
Age (years), mean ± SD	53.9 ± 15.5
Female sex, *n* (%)	19 (86.4%)
Male sex, *n* (%)	3 (13.6%)
Right foot, *n* (%)	16 (53.3%)
Left foot, *n* (%)	14 (46.7%)
Second intermetatarsal space, *n* (%)	8 (26.7%)
Third intermetatarsal space, *n* (%)	22 (73.3%)
Ultrasound-measured neuroma size. mean ± SD (range) (mm)	5.96 ± 1.52 (3.6–9.7)

**Table 2:** Clinical outcomes during follow-up.

Variable	Preoperative	3 Months	6 Months	12 Months
Pain Visual Analogue Scale, mean ± SD	8.67 ± 1.35	1.03 ± 1.43	0.93 ± 1.57	1.67 ± 2.86
(*p* from baseline)		<0.001	<0.001	<0.05
Foot Function Index (%), mean ± SD	42.3 ± 13.4	—	5.0 ± 7.4	8.0 ± 16.2
(*p* from baseline)			<0.001	<0.05
Giannini score, mean ± SD	—	—	—	68.86 ± 18.53
Neuromas with Giannini score ≥60, *n* (%)	—	—	—	26 (86.7%)

**Table 3:** Comparison of outcomes across 8 studies with radiofrequency for Morton’s neuroma.

Author	*n*	Technique	Follow-Up (Months)	Success Rate	Complications
Moore et al. [14]	32	CRF · 85 °C · 90 s	13	83% (24/29) complete relief	1 superficial cellulitis
Chuter et al. [13]	30	CRF · 81 °C · 5 × 2 min	6	86.7% satisfactory outcome	1 transient posterior tibial nerve irritation
Genon et al. [32]	38	CRF · 90 °C · 3 min	10.6	73.7% complete/partial relief; 86.8% satisfied	0% RF-related
Deniz et al. [33]	22	PRF · 42 °C · 300 s	9	60% achieved ≥50% pain relief	10% minor
Brooks et al. [18]	32	CRF· 90 °C · 4 min × 2–3 cycles	12.9	87.5% very/moderately satisfied	0%
Masala et al. [15]	52	CRF · 85 °C · 90 s	12	88.5% durable relief (46/52) · VAS 9.0 → 2.0	0%
Shah et al. [34]	22	CRF · 80 °C · 3–10 × 1 min	8	89% satisfied	0% major
Present study	30	CRF · 85 °C + neurophysiological monitoring	12	86.7% (96.3% adjusted)	0% major

Note: The highlighted row (blue) indicates the present study. CRF = continuous radiofrequency; PRF = pulsed radiofrequency.

## Data Availability

The data supporting the findings of this study are available from the corresponding: authors upon reasonable request. Due to privacy and ethical restrictions, the data are not publicly deposited.

## Abbreviations

The following abbreviations are used in this manuscript:

FFI	Foot Function Index
Giannini score	Giannini Interdigital Neuroma Clinical Evaluation Score
IQR	Interquartile Range
NAPs	Nerve Action Potentials
RF	Radiofrequency
SD	Standard Deviation
SNAPs	Sensory Nerve Action Potentials
VAS	Visual Analog Scale
